# Supplementary material for: Signatures of paracrystallinity in amorphous silicon from machine-learning-driven molecular dynamics
Source: Nat Commun. 2025 Mar 10;16:2360. doi: 10.1038/s41467-025-57406-4 (PMC11894222; doi:10.1038/s41467-025-57406-4)
Supplement: Supplementary file 1 — Supplementary Information [file 41467_2025_57406_MOESM1_ESM.pdf]

Supplementary Information for
‘Signatures of paracrystallinity in amorphous silicon from machine-learning-driven molecular dynamics’

Louise A. M. Rosset[1], David A. Drabold[2], and Volker L. Deringer*[1]

[1]Department of Chemistry, University of Oxford, Oxford, UK
[2]Department of Physics and Astronomy, Ohio University, Athens, OH, USA

*volker.deringer@chem.ox.ac.uk

Contents

1. Methods **S4**

A. Structure generation S4

1. Dataset S4

2. Large-scale models S5

B. Choice of potential S6

C. Structural analysis S8

1. Structural descriptors S8

2. Medium-range order S12

3. Structure factor S12

2. Results **S13**

A. Dataset S13

B. Continuous Random Networks S15

C. Range of paracrystallinity S16

1. 1,000-atom structures S16

2. Other system sizes S17

D. Clustering of ‘diamond’-like environments S18

E. Paracrystalline grain stability S20

Supplementary references **S21**

List of Figures

1 Molecular dynamics protocol. S4
2 Choice of anneal time. S5
3 Comparison of computational cost of different potentials. S6
4 Structure energy prediction comparison. S7
5 Local energy prediction comparison. S7
6 Structure relaxation prediction comparison. S8
7 Effect of PTM threshold on categorization. S10
8 Comparison of descriptors of diamond-like environment predictions. S11
9 Alternative structure factor calculation. S12
10 Dataset split by structure size contribution. S13
11 Dataset split by quench rate contribution. S14
12 Comparison of CRN models of 1,000 atoms. S15
13 Renders of the structures from Fig. 2. . . . . S16
14 Short range order of the structures from Fig. 2. . . . . S16
15 Paracrystalline structures of 216 and 512 atoms. S17
16 Number of clusters per structure in structures of 1,000 atoms. S18
17 Shortest distance between clusters in structures of 1,000 atoms. S19
18 Temperature stability of diamond-like environments. S20

List of Tables

1 Choice of PTM cutoff threshold. S9
2 Clustering analysis on size and number of clusters. S18
3 Clustering analysis on the connectivity of clusters. S19

Supplementary Note 1. Methods

A. Structure generation

1. Dataset

To build our dataset of silicon configurations, we follow the protocol depicted in Suppl. Fig. 1 using LAMMPS.[S1] We start with randomized hard-sphere structures of varying system size (64, 216, 512, and 1,000 atoms) and uniformly sample densities between 2.1 and 2.5 g cm^{-3} with a step size of 0.002 g cm^{-3}, chosen to provide a range of underdense and overdense a-Si structures. These structures are melted at 2,000 K for 10 ps, then quenched to 500 K at various quench rates from 10^{13} K/s to 10^{10} K/s in the canonical ensemble (NVT). Finally they are annealed for 10 ps at 300 K in the isothermal–isobaric ensemble (NPT). The final structure is added to the dataset. We employ a Nosé–Hoover thermostat for the NVT simulations and a Nosé–Hoover thermostat and barostat for the NPT simulations. These simulations are run with a timestep of 1 fs.

The melt-quench simulations of 1,000 atoms at rates of 10^{10} K/s for structures with densities $\rho > 2.29$ g cm^{-3} were omitted as they were computationally expensive and systematically resulted in polycrystalline structures with high counts of diamond-like environments, not highly relevant to our study of paracrystalline silicon, and we already had many such structures.

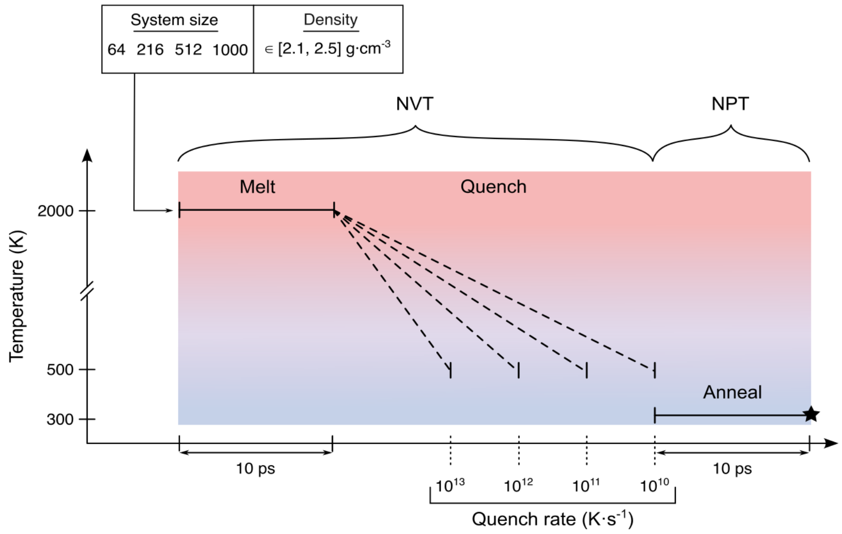

Supplementary Figure 1: Schematic overview of the protocol used for the molecular-dynamics simulations reported in the present work. The last frame from the simulation is indicated by a star; this frame is added to our dataset.

We investigated the stability of our generated structures by selecting 100 quenched structures at random from our dataset, and annealing them for 1 ns at 300K, instead of 10 ps from our protocol. We plot the energy and the structural similarity to c-Si of the structures for different annealing protocols in Suppl. Fig. 2.

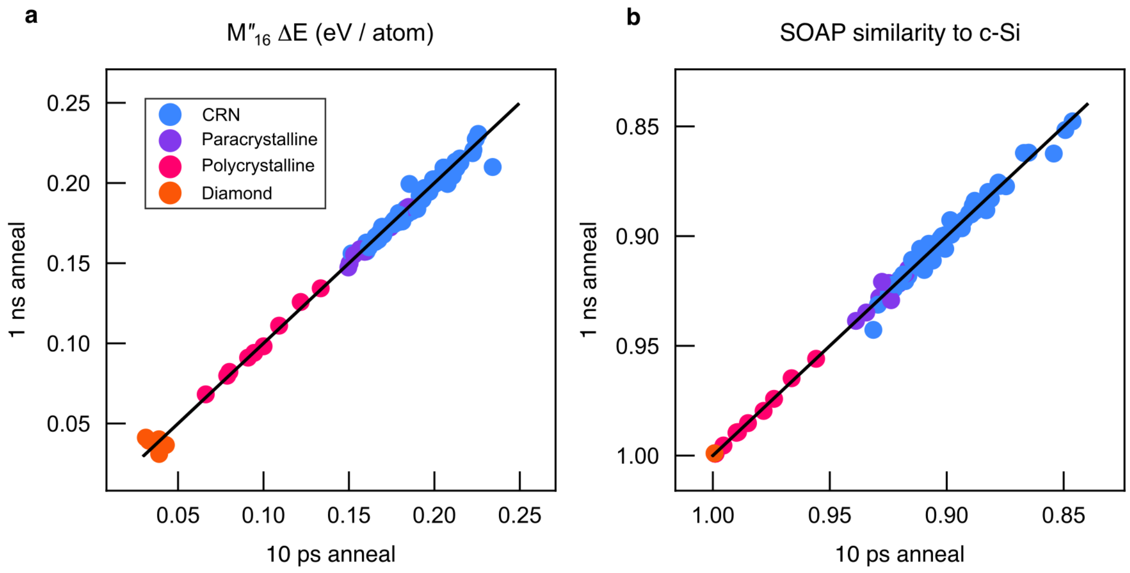

Supplementary Figure 2: Pairplots comparing a 10 ps anneal against a 1 ns anneal for 100 structures chosen at random from our dataset, assessed on (a) the MTP energy relative to c-Si and (b) the SOAP similarity to c-Si. Source data are provided as a Source Data file.

We observe that a longer anneal does not significantly affect the energetics or the topology of our structures. Only the distorted diamond structures (orange) show considerable change in energetics.

2. Large-scale models

The 100,000-atom structures with 0.8% (796 atoms) and 62.3% (62,275 atoms) of diamond-like environments were generated by quenching randomized cells of density $\rho = 2.252\ \mathrm{g\,cm^{-3}}$ at rates of 10^{11} and 10^{10} K/s respectively, following the same protocol as laid out in Suppl. Fig. 1. We substitute the anneal treatment for an NPT annealing run at 300 K for 50 ps with the Si-GAP-18 potential.[S2]

B. Choice of potential

We use the M''_{16} 'student' potential of Ref. S3 to drive our MD simulations. This potential, fitted using the Moment Tensor Potential (MTP) framework,[S4,S5] is two orders of magnitude faster than its 'teacher' counterpart, Si-GAP-18,[S2] as seen in Suppl. Fig. 3. The teacher–student approach therefore allows us to use M''_{16} to simulate quench rates as slow as 10^{10} K/s, which are not easily accessible for slow quenches of large system sizes using the teacher model.

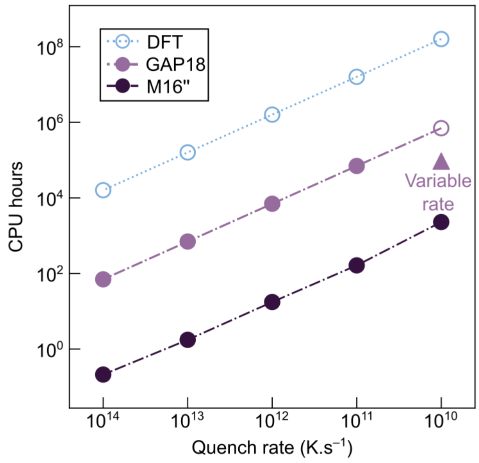

Supplementary Figure 3: Comparison of the computational cost of quench simulations of a 512-atom system at different quench rates using DFT-, Si-GAP-18- ('GAP18') and M''_{16}-driven MD, drawn similar to Ref. S6. A triangle indicates the variable rate protocol using GAP from Ref. S7, which we reproduced to determine the computational cost. DFT computational time estimates are taken from Ref. S6. Source data are provided as a Source Data file.

To verify the energy predictions by the M''_{16} potential, we first compare the energy predictions, relative to c-Si, for all structures in the dataset, by M''_{16} to those by the Si-GAP-18[S2] and by Si-ACE-21 potentials[S8]. The predictions and overall trends computed with M''_{16}, shown in Suppl. Fig. 4, are in line with the results of both Si-GAP-18 and Si-ACE-21.

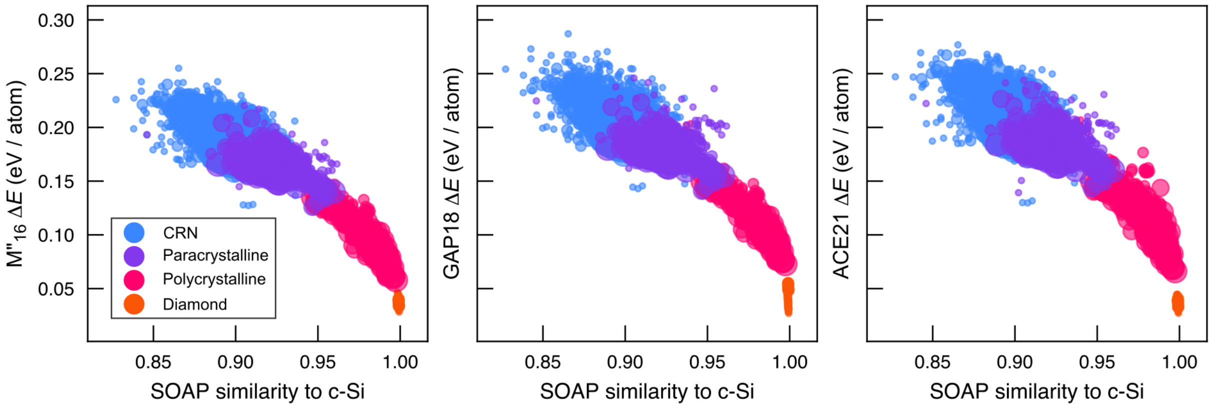

Supplementary Figure 4: Comparison of the structure energy prediction by the M''_{16} (*left*), Si-GAP-18 (*middle*) and Si-ACE-21 (*right*) potentials for all structures in the dataset, as analyzed in Fig. 1. Source data are provided as a Source Data file.

We also compare the local-energy predictions, averaged over nearest neighbors, by M''_{16} to those by Si-GAP-18 and Si-ACE-21, shown in Suppl. Fig. 5. Again, the predictions and overall trends computed with M''_{16}, agree with the results of both Si-GAP-18 and Si-ACE-21.

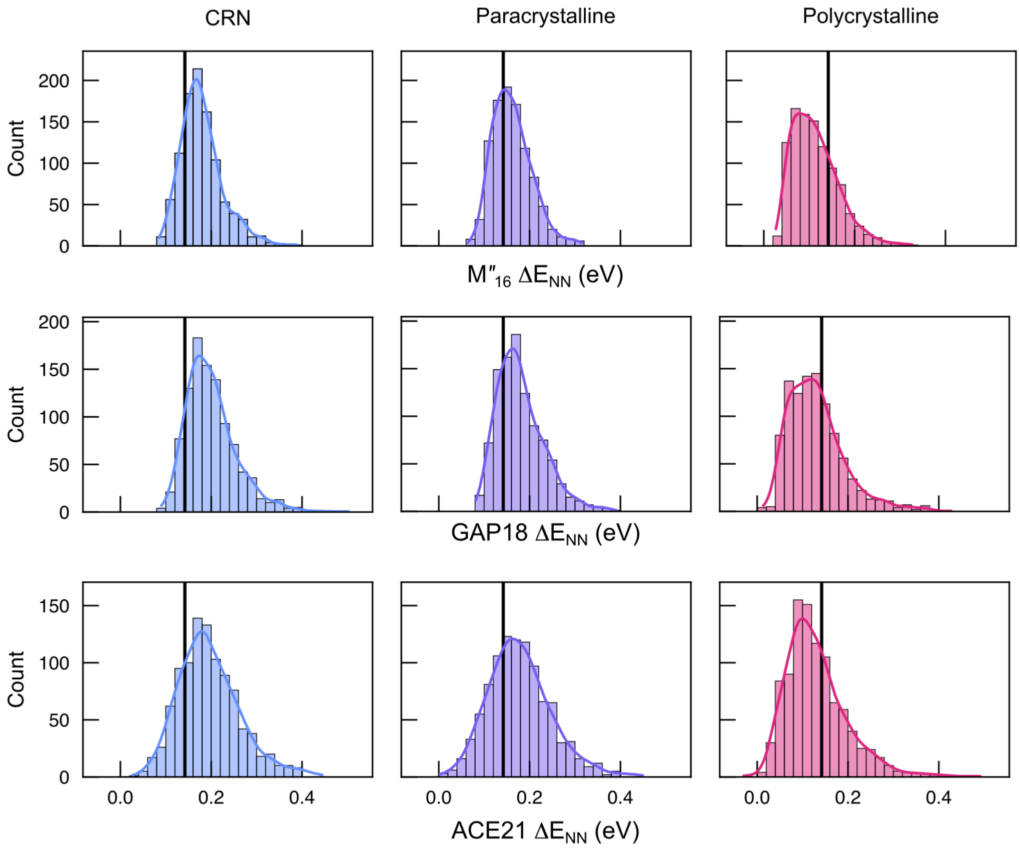

Supplementary Figure 5: Comparison of the local energy prediction by the M''_{16} (*top row*), Si-GAP-18 (*middle row*) and Si-ACE-21 (*bottom row*) potentials for the structures analyzed in Fig. 4. Source data are provided as a Source Data file.

We assess the local minima predictions of the three potentials by relaxing 100 quenched structures chosen at random from our dataset, to a force stopping tolerance of 10^{-6} eV/Å. We plot the energies for each relaxed structure in Suppl. Fig. 6. The models show qualitative agreement, although the M''_{16} potential over-stabilizes the highly distorted CRN structures.

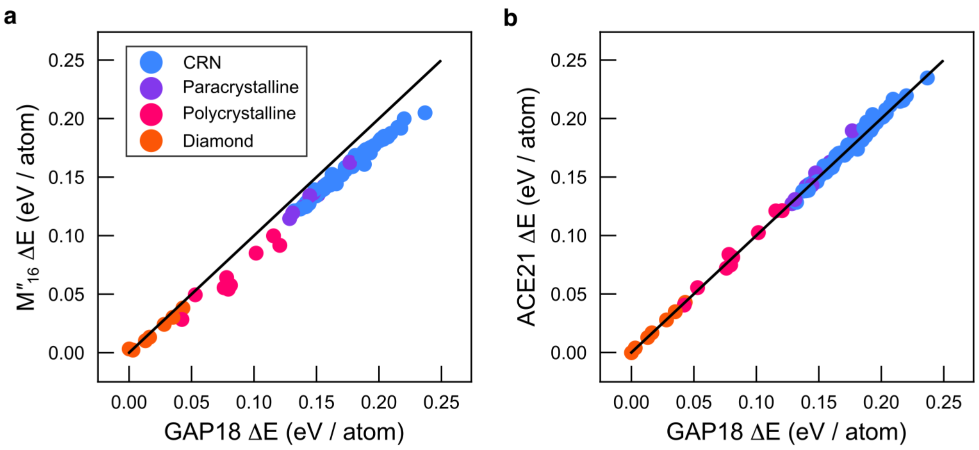

Supplementary Figure 6: Pair plots comparing the relative energy of 100 structures chosen at random from our dataset, relaxed with the Si-GAP-18 compared to a relaxation with (a) M''_{16} and (b) Si-ACE-21. Source data are provided as a Source Data file.

Finally, for an additional check, we quench an additional 100,000-atom structure using the Si-ACE-21 potential at a quench rate of 10^{11} K/s, following the protocol in Section A.2. of this Supplementary Note. We obtain an annealed structure with 0.3%, or 309 atoms, in diamond-like environments—similar to the 100,000-atom structures obtained with Si-GAP-18 (0.3%, or 273 atoms; Ref. S9) and M''_{16} (0.8%, or 796 atoms).

C. Structural analysis

1. Structural descriptors

Polyhedral Template Matching. We use Polyhedral Template Matching (PTM)[S10] as implemented in OVITO[S11] to identify the local crystalline structure of atomic environments. This classification method matches the atomic neighborhood of an atom to templates of different crystalline structures using convex hulls, and assigns crystalline structure types within a deviation metric (RMSD cutoff), or the type 'other' beyond the cutoff. We choose a RMSD cutoff of 0.1 for our analysis at 300 K, in accordance with Ref. S10.

Both cubic diamond (**dia**) and hexagonal diamond (**lon**) templates contain four first-neighbor atoms and eight second-neighbor atoms. The structure types differ by the conformation of the

6-membered rings—which is ‘chair’ for **dia** and a combination of ‘chair’ and ‘boat’ for **lon**. The majority of neighborhoods identified as **lon**-like environments are located at grain boundaries, grain–matrix boundaries, or are stacking defects. This assignment to **lon** rather than **dia** could be an artefact of the template method.

We then assign each structure to a category depending on the proportion of locally ‘crystal-like’ atoms identified by PTM, as described in the Methods section of the main text.

We investigate the change in the count and distribution of the paracrystalline structures for varying thresholds in Tab. 1. At our chosen threshold of 15%, around 75% of the structures in the ‘paracrystalline’ category have 5% of diamond-like environments or fewer. These are the structures that our analysis focuses on in Fig. 2. Increasing the threshold further does not cause a sharp increase in the number of identified paracrystalline structures. We argue that while the threshold is important, a choice of 5, 10, 15 or 20 % does not significantly change the analysis. Our dataset has a deficit in structures with local crystallinity between 15 and 40%, with a change of only around 3.2 points when changing the threshold from 15 to 40%.

Supplementary Table 1: Effect of the choice of PTM cutoff threshold (%) for the maximum number of ‘diamond’-like atoms in a structure on the count of paracrystalline structures in the dataset, and on the average distribution of these structures. Source data are provided as a Source Data file.

Polyhedral Template Matching threshold (%)	% of paracrystalline structures in dataset	SOAP sim. to c-Si		ΔE rel. to c-Si (eV)	
		Mean	SD	Mean	SD
1	3.9	0.917	0.011	0.170	0.011
2	7.0	0.919	0.012	0.170	0.013
3	8.0	0.920	0.011	0.169	0.012
4	8.9	0.921	0.011	0.168	0.012
5	9.4	0.921	0.012	0.168	0.012
10	11.1	0.923	0.012	0.166	0.013
15	12.7	0.926	0.014	0.165	0.013
20	13.6	0.927	0.014	0.164	0.013
25	14.4	0.928	0.015	0.163	0.014
30	15.0	0.930	0.016	0.162	0.015
35	15.5	0.931	0.016	0.161	0.016
40	15.9	0.932	0.017	0.159	0.017
45	16.7	0.933	0.019	0.157	0.020
50	17.1	0.934	0.020	0.156	0.021

We show the effect of increasing the threshold on the distribution from Fig. 1 of the main text in Suppl. Fig. 7. As an inset for each plot, we include the most extreme structure labeled as ‘paracrystalline’ at that threshold. In Suppl. Fig. 7a, the most extreme structure at a threshold

of 10% contains isolated clusters, while the most extreme structure at 15% is formed of one cluster. At 20%, the cluster has grown larger. At high thresholds, the structures transition from a CRN matrix with a few diamond-like environments to a mixed phase with large grains (Suppl. Fig. 7b).

We choose a threshold of 15% as a limit between the 'paracrystalline' and 'polycrystalline' categories as we see clustering of the diamond-like environments beyond 15%, leading to structures with true grains rather than isolated crystalline environments.

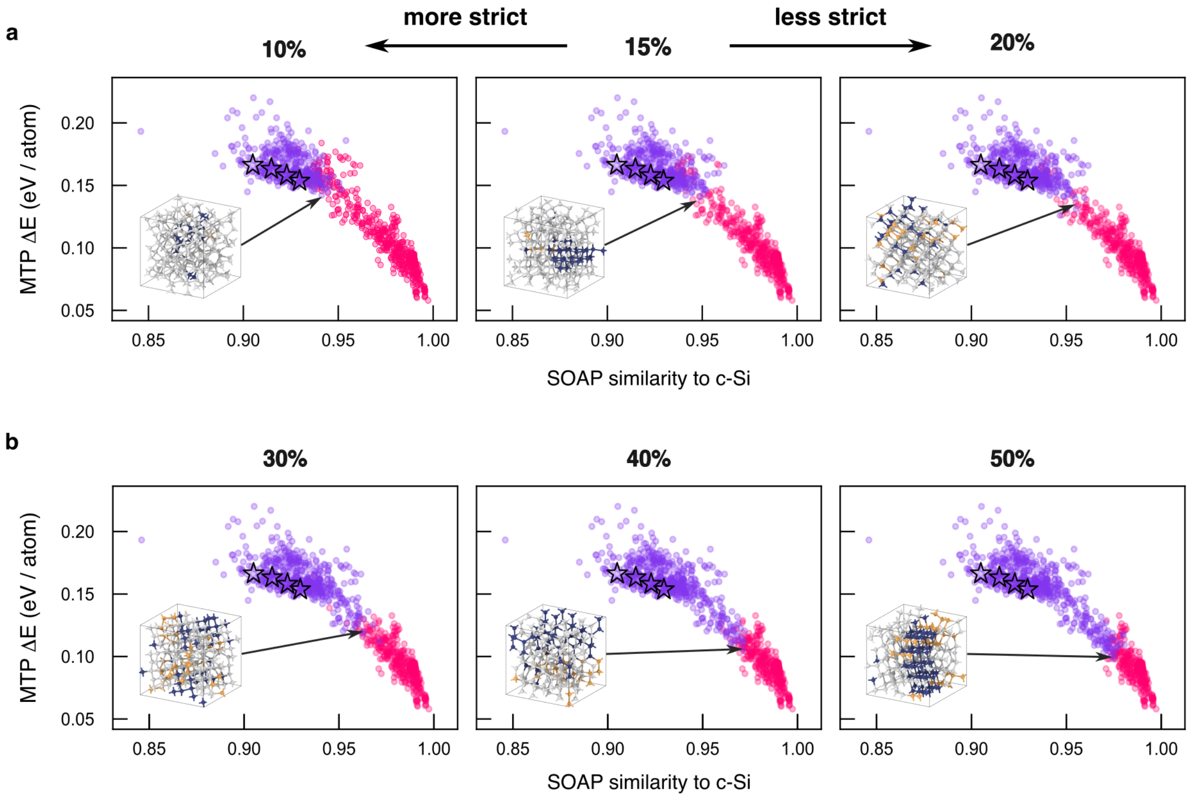

Supplementary Figure 7: (a) Plot of the distribution of paracrystalline (purple) and polycrystalline (pink) structures for varying PTM thresholds, alongside a render of the most extreme paracrystalline structure, generated using OVITO.[S11] (b) We indicate the structures **I** to **IV**, studied in Fig. 2, with stars. Source data are provided as a Source Data file.

Common Neighbor Analysis. We use Common Neighbor Analysis (CNA) to identify neighborhoods around crystalline environments.[S11–S13] While CNA is less reliable than PTM[S10] as it uses Euclidean distances to match environments to structure types, it provides information about the neighborhoods around identified crystalline-like atomic environments.

SOAP similarity to c-Si. We compute SOAP descriptors for our entire structures and for atomic environments using the QUIP package[S14]. We then obtain the SOAP similarity to c-Si using: $(q_{env} \cdot q_{dia})^{\zeta}$, where q_{env} is the computed SOAP vector, q_{dia} is the SOAP vector of a c-Si environment and $\zeta = 4$.

Bond orientational order parameters. We compute bond orientational order (BOO) descriptors for atomic environments using the implementation from Ref. S15, with $r_{\text{cut}} = 2.85$, $n_{\text{neigh}} = 16$, $ql_{\text{values}} = [3-13:1]$, $wl_{\text{values}} = [4-14:2]$. We then obtain a BOO similarity to c-Si using $(q_{\text{env}} \cdot q_{\text{dia}})$, where q_{env} is the computed BOO descriptor and q_{dia} is the BOO descriptor of a c-Si environment.

Comparison of structural descriptors. We compare the identification of diamond-like environments in paracrystalline and polycrystalline structures using the four structural descriptors presented above. We present the results in Suppl. Fig. 8, using a correlation heatmap.

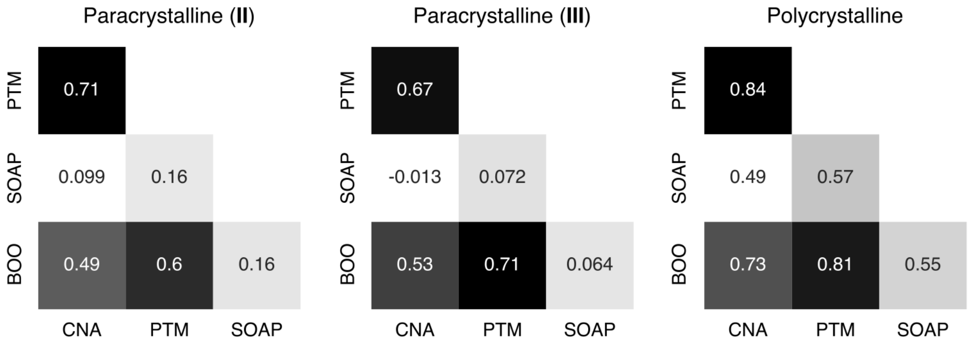

Supplementary Figure 8: Correlation heatmap of the prediction of diamond-like environments for four descriptors: Polyhedral Template Matching (PTM) with a cutoff of 0.1, Common Neighbor Analysis (CNA), SOAP similarity to c-Si with cutoffs of 0.99, 0.99 and 0.97 respectively, and the BOO similarity to c-Si with cutoffs of 0.05, 0.04 and 0.03 respectively. Source data are provided as a Source Data file.

While both PTM and CNA provide a straightforward definition of a 'diamond-like' environment, we must define a threshold for diamond-likeness for the SOAP and BOO similarity measures. Hence for each structure, we choose the SOAP and BOO similarity thresholds that provide the best agreement to the other descriptors.

It is important to note that the maximum BOO similarity value changes greatly from one structure to another, respectively 0.061, 0.068 and 0.046 for the structures presented in Suppl. Fig. 8. This means choosing a single threshold for all structures is impossible, and the threshold for

diamond-likeness changes even within the same category of structures: 0.05 for **II** vs 0.04 for **III** to obtain good agreement with CNA and PTM.

Across the three structures, the SOAP similarity to c-Si consistently provides the worst agreement with the other methods. The BOO similarity and CNA perform similarly, while the PTM provides the best agreement and the most straightforward implementation.

2. Medium-range order

For the analysis of the medium-range order characteristics in Fig. 2., we chose four paracrystalline structures of increasing paracrystallinity from the set of structures of 1,000 atoms and generated by melt-quenching at a rate of 10^{11} K/s for consistency in the structural quality of these models. Similarly, the CRN chosen for comparison is also from a 10^{11} K/s quench.

3. Structure factor

We computed the structure factor in an alternative way, via the Fourier transform of the radial distribution function, as shown in Suppl. Fig. 9. This method is directly comparable to the experiment, without need for normalization.

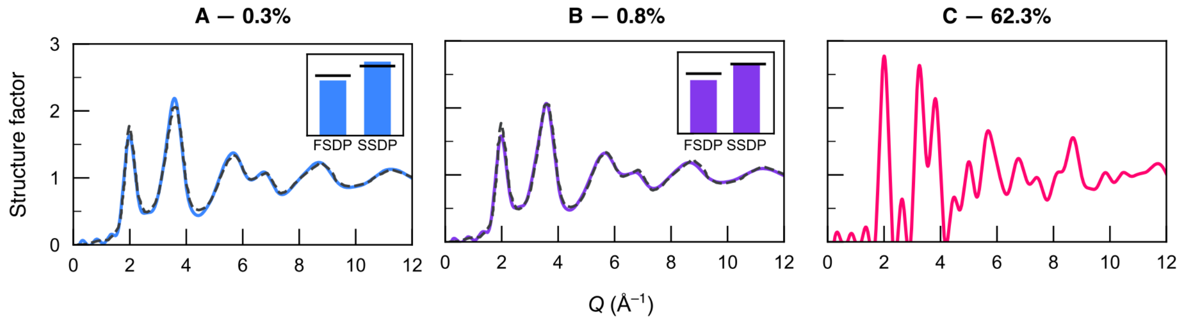

Supplementary Figure 9: Computed structure factor, as obtained via the Fourier transform of the radial distribution function, for each structure presented in Fig. 5a of the main text, from low paracrystallinity [structures **A** (*left*) and **B** (*middle*)] to polycrystallinity [structure **C** (*right*)]. Black dashed lines indicate the experimental data from Ref. S16. The insets show the agreement of the first and second sharp diffraction peaks with the experimental data (black lines). Source data are provided as a Source Data file.

Supplementary Note 2. Results

A. Dataset

To visualize the effect of different simulation-cell sizes in the dataset, we plot the contribution of each cell size to the total dataset in Suppl. Fig. 10. Here, we plot the contributions from individual atomic environments. The 64-atom cells are the only ones that fully crystallize (orange markers at a SOAP similarity close to 1). The 216-, 512-, and 1,000-atom cells all have similar contributions to the dataset in terms of the structural diversity covered.

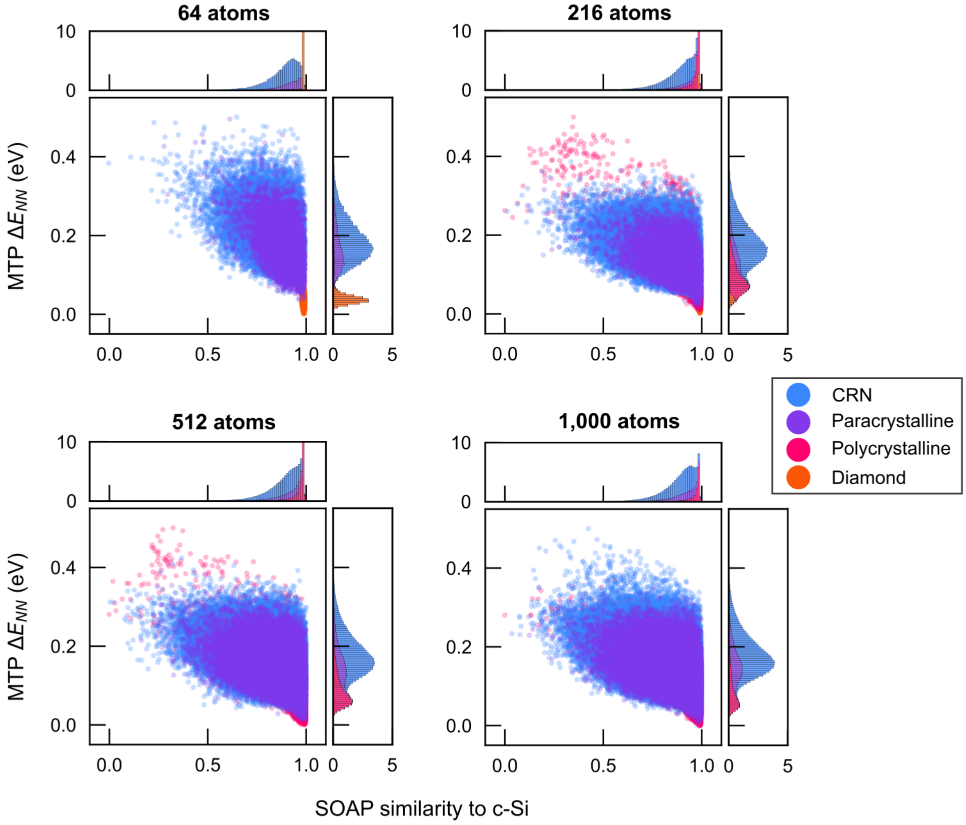

Supplementary Figure 10: Characterization of the individual atomic environments in the total dataset, plotted by cell-size contribution with 801 structures of 64 atoms per cell, 782 structures of 216 atoms per cell, 797 structures of 512 atoms per cell, and 689 structures of 1,000 atoms per cell. Stacked histograms of the SOAP similarity to c-Si (top) and per-atom energies (right) are shown using the same axes as the scatter plot, shown as percentage values of the total cell-size contribution. Source data are provided as a Source Data file.

In Suppl. Fig. 11, we characterize the dataset using separate plots for each quench rate. Partial and full crystallization occurs at quench rates of 10^{10} and 10^{11} K/s. Even at the same quench rate, structures of different densities can amorphize or crystallize.

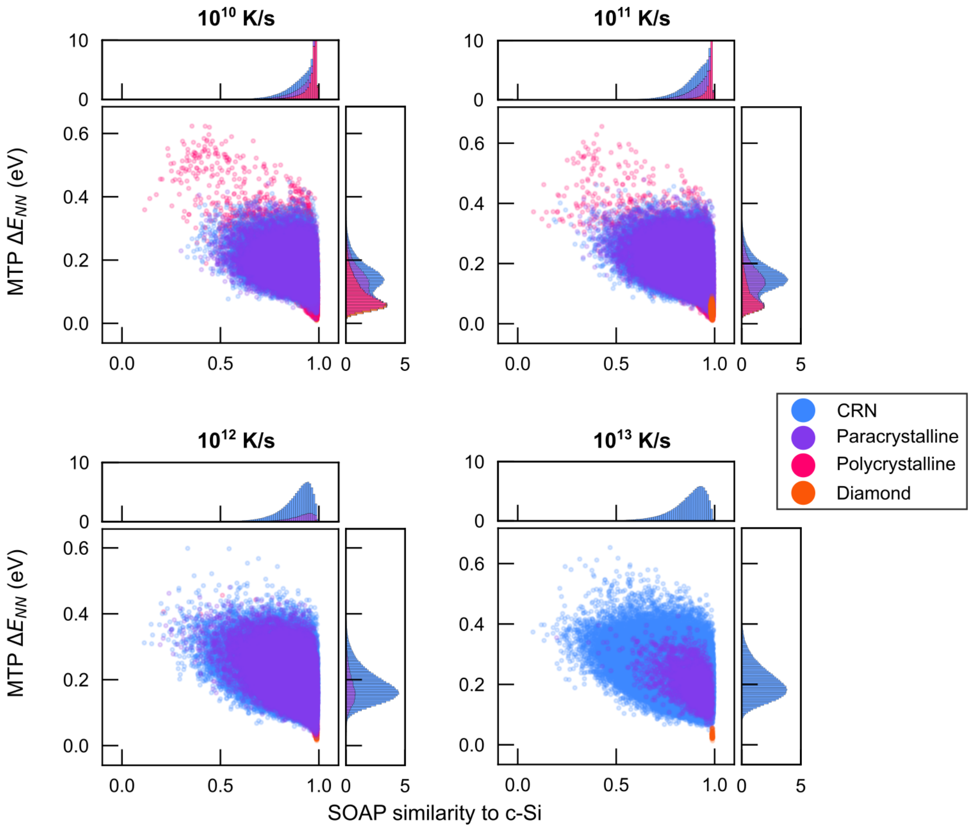

Supplementary Figure 11: As Suppl. Fig. 10, but now showing the results separately according to the quench rate. Source data are provided as a Source Data file.

B. Continuous Random Networks

We compare four structures of 1,000 atoms from the 'CRN' category to investigate the structural diversity within the CRNs in our dataset. In particular, we choose two CRN structures of essentially the same SOAP similarity (= 0.899) in red and yellow in Suppl. Fig. 12 a), and two structures of the essentially same energy ($\Delta E = 0.205$ eV) in green and blue, and investigate their medium-range order characteristics in Suppl. Fig. 12b. Importantly, we could only choose structures generated from melt-quench simulations at the same quench rate to ensure that there is no influence due to the speed of the quench. Here we choose a quench rate of 10^{13} K/s, as it provides the largest diversity of environments.

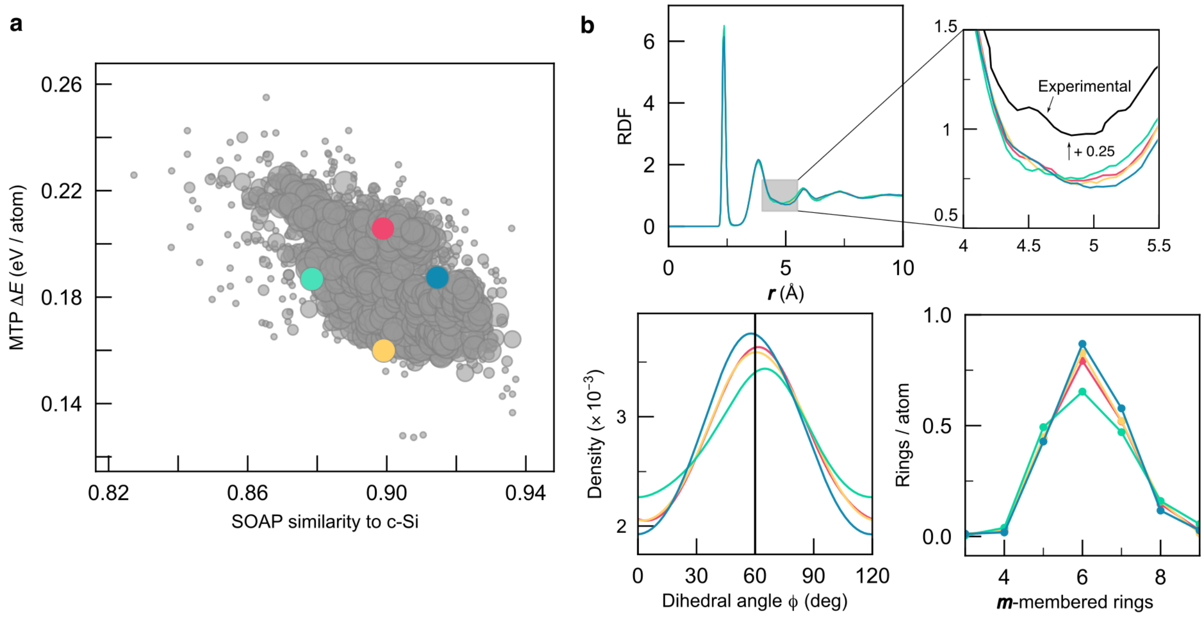

Supplementary Figure 12: Comparison of CRN models of 1,000 atoms. (a) Map of similarity to diamond-type Si against the predicted excess energy for CRN structures only, where four selected structures have been colored. (b) Characteristics of medium-range order for the four selected structures. Source data are provided as a Source Data file.

The radial distribution functions of the four CRNs are very similar and show no enhancement between the second and third peaks. They show some variation in both the dihedral angle and ring distributions, but within a smaller range than that of the paracrystalline studied in Fig. 2. The structure indicated in green is noticeably different from the others, which is likely due to its low density of $\rho = 2.152$ g cm^{-3} compared to the others. Some of the variation in the medium-range order can also be attributed to the fast quench rate.

C. Range of paracrystallinity

1. 1,000-atom structures

The structures investigated in Fig. 2 and depicted in Suppl. Fig. 13 present little variation in their bond-angle distribution and short-range order, as shown in Suppl. Fig. 14.

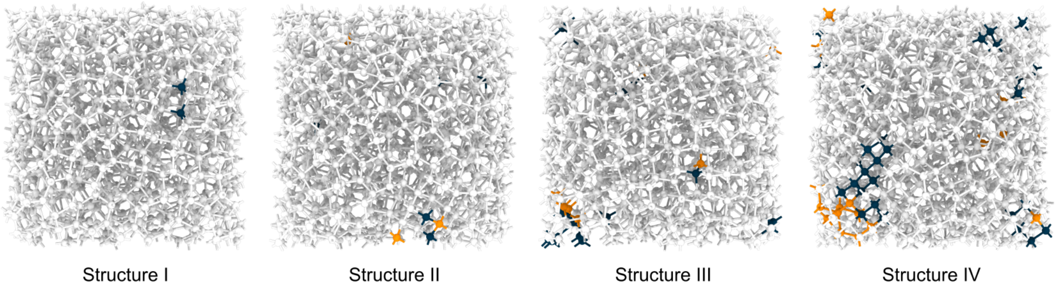

Supplementary Figure 13: Visualization of the four paracrystalline structures analyzed in Fig. 2, using OVITO.[S11]

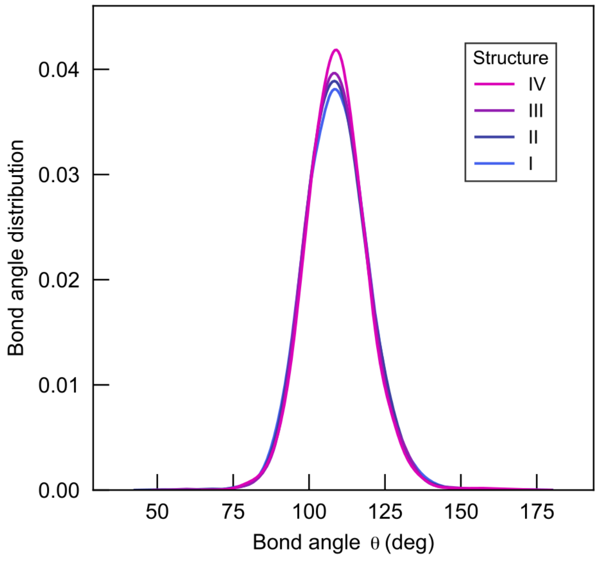

Supplementary Figure 14: Bond-angle distribution of the four paracrystalline structures analyzed in Fig. 2. Source data are provided as a Source Data file.

2. Other system sizes

We replicate the study of paracrystallinity on the other system sizes in our dataset, viz. 216 and 512 atoms, in Suppl. Fig. 15. We omit the structures of 64 atoms per cell, as they are too small for meaningful radial distribution functions.

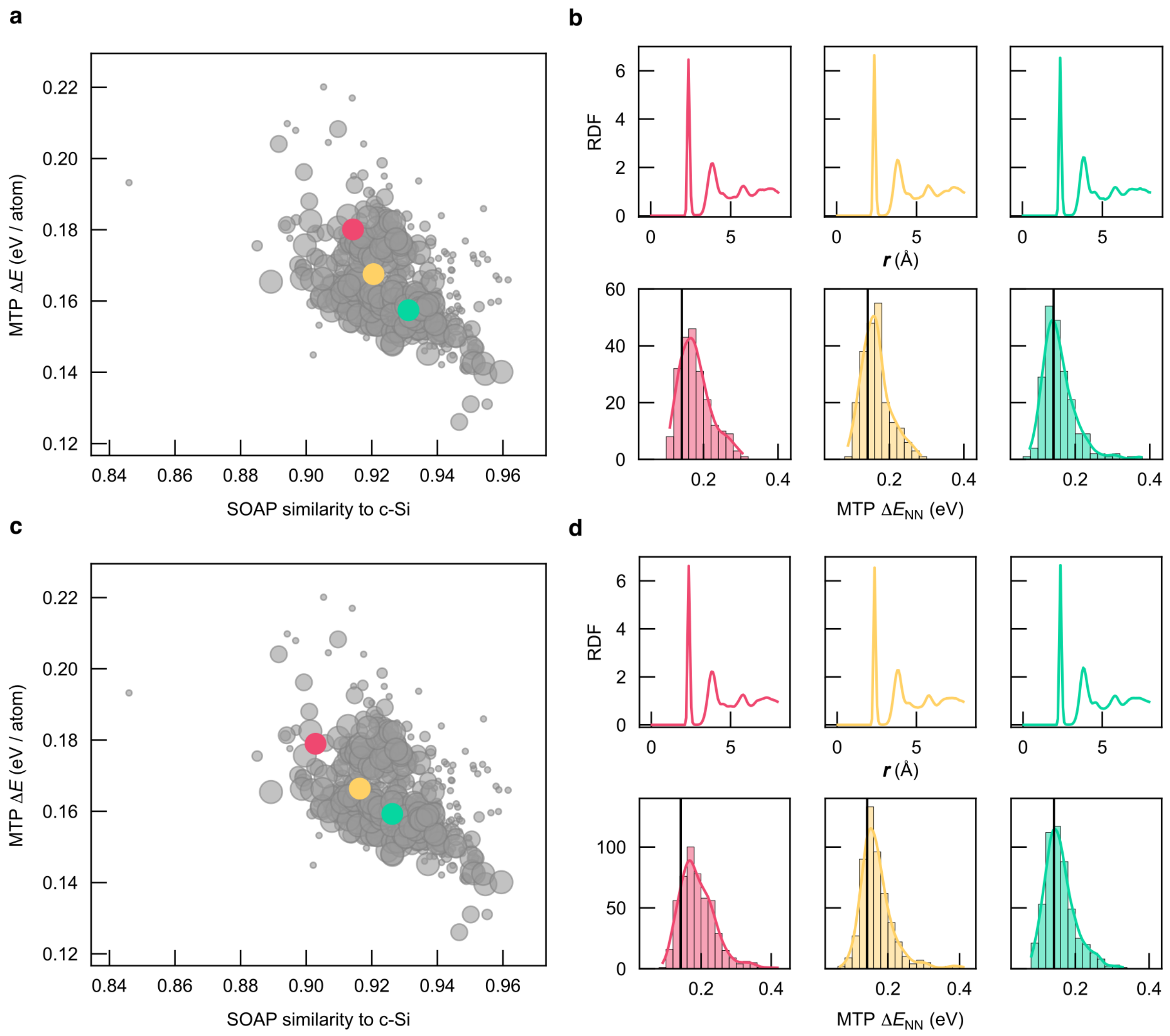

Supplementary Figure 15: A study of paracrystalline structures of 216 atoms (top) and 512 atoms (bottom). Three paracrystalline structures of increasing paracrystallinity are chosen from the set of paracrystalline structures, highlighted in (a). Their radial distribution function and energetics are presented in (b). The same protocol is applied for structures of 512 atoms in (c) and (d). Source data are provided as a Source Data file.

We see that these smaller structures reproduce our findings, that paracrystallinity influences medium-range order and tends to lower the total energy.

D. Clustering of ‘diamond’-like environments

Here we provide the full statistics to complement Fig. 3. We provide an alternative calculation of the distance between clusters in Table 3: the shortest distance between any two atoms in two clusters, and the distance between the centers of mass of the clusters.

Supplementary Table 2: Analysis of size and occurrence of diamond-like environments in structures of 1,000 atoms. Source data are provided as a Source Data file.

SOAP sim. to c-Si	# of structures	Total # of clusters	# of clusters per structure		# of atoms per cluster	
			Mean	SD	Mean	SD
0.88	8	8	1.00	0	1.1	0.33
0.89	34	43	1.26	0.56	1.3	0.5
0.90	74	119	1.61	1.09	1.3	0.7
0.91	99	229	2.31	1.64	1.6	1.9
0.92	99	369	3.73	1.67	2.2	2.4
0.93	51	309	6.06	3.09	4.8	5.7
0.94	11	87	7.91	3.00	8.0	4.3
0.95	10	55	5.50	2.94	63.7	57.5
0.96	5	22	4.40	2.58	91.8	53.9
0.97	15	44	2.93	1.44	198.7	116.1
0.98	28	50	1.79	1.11	459.1	195.7
0.99	36	48	1.33	0.88	726.2	216.1
1	5	5	1.00	0	930.4	14.54

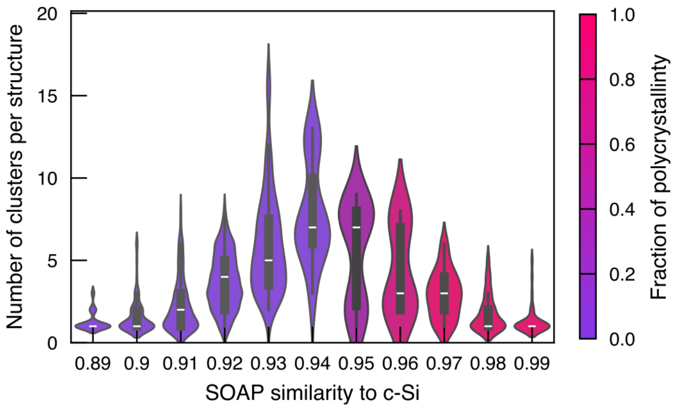

Supplementary Figure 16: Number of clusters per structure in structures of 1,000 atoms, presented as violin plots where the boxes show the interquartile range (IQR), the median is shown as a white line, and the whiskers show the range of the distribution within 1.5× IQR. We use a kernel bandwidth of 1 for the kernel density estimate. Source data are provided as a Source Data file.

Supplementary Table 3: Analysis of the connectivity of diamond-like environments in structures of 1,000 atoms. Source data are provided as a Source Data file.

SOAP sim. to c-Si	Shortest distance between clusters (Å)		Distance between cluster centers (Å)	
	Mean	SD	Mean	SD
0.89	7.2	3.6	5.3	4.8
0.90	10.7	4.0	9.6	7.8
0.91	10.9	3.6	13.4	9.8
0.92	11.4	3.1	17.6	10.2
0.93	12.8	2.2	32.0	19.1
0.94	12.8	0.5	48.1	16.8
0.95	13.5	2.3	32.0	17.2
0.96	15.7	1.9	28.1	16.3
0.97	12.0	3.1	16.4	8.1
0.98	12.0	3.2	11.6	5.1
0.99	13.3	2.1	14.0	6.1

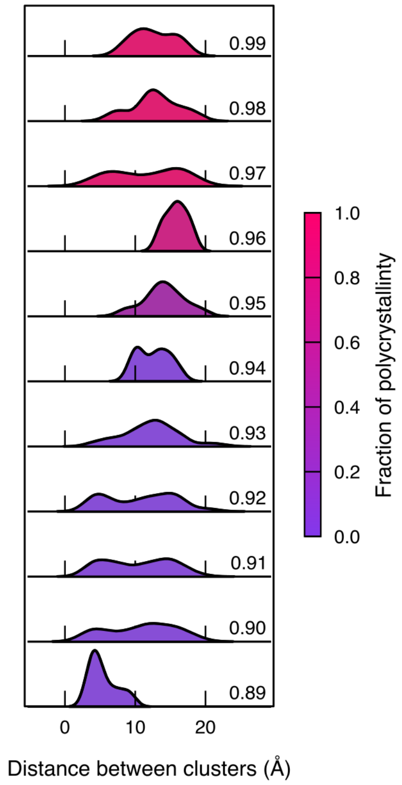

Supplementary Figure 17: Shortest distance between clusters in structures of 1,000 atoms. Source data are provided as a Source Data file.

E. Paracrystalline grain stability

To evaluate the stability of our paracrystalline structures and to determine whether the local ordering could be the onset of crystallization, we carried out high-temperature annealing simulations for the structures presented in Fig. 5. The structures were held at temperatures ranging from 1,000 to 1,500 K for 1 ns in the NPT ensemble. This protocol was repeated five times at each temperature. The evolution of the percentage of diamond-like environments, both **dia** and **lon**, is plotted as a function of the progress of the simulation. At temperatures below 1,200 K, diffusion is limited and the count of diamond environments is stable. At 1,200 K, sufficient energy is supplied for diffusion and both the CRN and paracrystalline structures see the nucleation and growth of a crystalline grain, although the onset of crystallization of the grain is earlier in the case of the paracrystalline model than it is for the CRN. The polycrystalline structure is stable at this temperature. For all annealing simulations at $T > 1{,}300$ K, the structures melt and the count of diamond-like environments drops to zero.

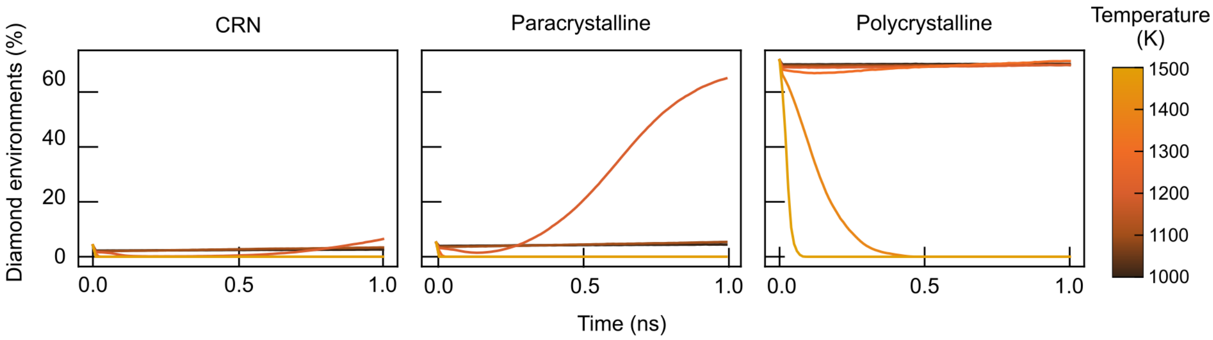

Supplementary Figure 18: Evolution of the count of diamond-like environments during high-temperature annealing simulations for the structures presented in Fig. 5 of the main text. Source data are provided as a Source Data file.

Supplementary references

S1. Thompson, A. P. *et al.* LAMMPS - a flexible simulation tool for particle-based materials modeling at the atomic, meso, and continuum scales. *Comp. Phys. Comm.* **271**, 108171 (2022).

S2. Bartók, A. P., Kermode, J., Bernstein, N. & Csányi, G. Machine Learning a General-Purpose Interatomic Potential for Silicon. *Phys. Rev. X* **8**, 041048 (2018).

S3. Morrow, J. D. & Deringer, V. L. Indirect learning and physically guided validation of interatomic potential models. *J. Chem. Phys.* **157**, 104105 (2022).

S4. Shapeev, A. V. Moment Tensor Potentials: A Class of Systematically Improvable Interatomic Potentials. *Multiscale Model. Simul.* **14**, 1153–1173 (2016).

S5. Novikov, I. S., Gubaev, K., Podryabinkin, E. V. & Shapeev, A. V. The MLIP package: Moment tensor potentials with MPI and active learning. *Mach. Learn.: Sci. Technol.* **2**, 025002 (2020).

S6. Deringer, V. L. *et al.* Realistic Atomistic Structure of Amorphous Silicon from Machine-Learning-Driven Molecular Dynamics. *J. Phys. Chem. Lett.* **9**, 2879–2885 (2018).

S7. Bernstein, N. *et al.* Quantifying chemical structure and machine-learned atomic energies in amorphous and liquid silicon. *Angew. Chem. Int. Ed.* **58**, 7057–7061 (2019).

S8. Lysogorskiy, Y. *et al.* Performant Implementation of the Atomic Cluster Expansion (PACE) and Application to Copper and Silicon. *npj Comput. Mater.* **7**, 97 (2021).

S9. Deringer, V. L. *et al.* Origins of structural and electronic transitions in disordered silicon. *Nature* **589**, 59–64 (2021).

S10. Larsen, P. M., Schmidt, S. & Schiøtz, J. Robust structural identification via polyhedral template matching. *Model. Simul. Mater. Sci. Eng.* **24**, 055007 (2016).

S11. Stukowski, A. Structure identification methods for atomistic simulations of crystalline materials. *Model. Simul. Mater. Sci. Eng.* **20**, 045021 (2012).

S12. Honeycutt, J. D. & Andersen, H. C. Molecular dynamics study of melting and freezing of small Lennard-Jones clusters. *J. Phys. Chem.* **91**, 4950–4963 (1987).

S13. Maras, E., Trushin, O., Stukowski, A., Ala-Nissila, T. & Jónsson, H. Global transition path search for dislocation formation in Ge on Si(001). *Comput. Phys. Commun.* **205**, 13–21 (2016).

S14. Kermode, J. R. f90wrap: an automated tool for constructing deep Python interfaces to modern Fortran codes. *J. Phys. Condens. Matter* **32**, 305901(2020).

S15. Faure Beaulieu, Z., Deringer, V. L., Martelli, F. High-Dimensional Order Parameters and Neural Network Classifiers Applied to Amorphous Ices. *J. Chem. Phys.* **160**, 081101 (2024).

S16. Laaziri, K. *et al.* High Resolution Radial Distribution Function of Pure Amorphous Silicon. *Phys. Rev. Lett.* **82**, 3460–3463 (1999).
